# Dissociating external and internal attentional selection

**DOI:** 10.1016/j.isci.2025.112282

**Published:** 2025-03-25

**Authors:** Kabir Arora, Surya Gayet, J. Leon Kenemans, Stefan Van der Stigchel, Samson Chota

**Affiliations:** 1 Helmholtz Institute, Utrecht University, Heidelberglaan 1, 3584CS Utrecht, the Netherlands

**Keywords:** Cognitive neuroscience, Psychology

## Abstract

Just as attention can shift externally toward relevant objects in the visual environment, it can shift internally toward relevant items within Visual Working Memory (VWM). Recent work has shown that spatial attention is automatically directed toward the previous location of an attended memory item, as it is to locations of perceived stimuli. When attending memory items, however, there is no sensory information to be processed at the previous location. Thus, we asked whether internal attention—akin to external attention—modulates sensory processing. In two EEG experiments, we compared location-specific sensory enhancements during attentional selection of external (perceived) versus internal (memorized) stimuli. Alpha-power and gaze-position biases confirmed an inherent spatial organization within VWM. However, Rapid Invisible Frequency Tagging (RIFT) revealed sensory modulation only during external attentional selection. Thus, VWM is not blindly recruiting existing mechanisms of external attention, but instead uses space as an organizational principle to store and select memories.

## Highlights

• Both internal and external attention involve shifts of spatial attention
• But sensory processing is only modulated during external, not internal attention
• VWM uses a spatial layout as a functional tool, distinctly from external attention
• RIFT responses do not correlate with alpha lateralization or small gaze shifts

## Graphical abstract

**Figure:**
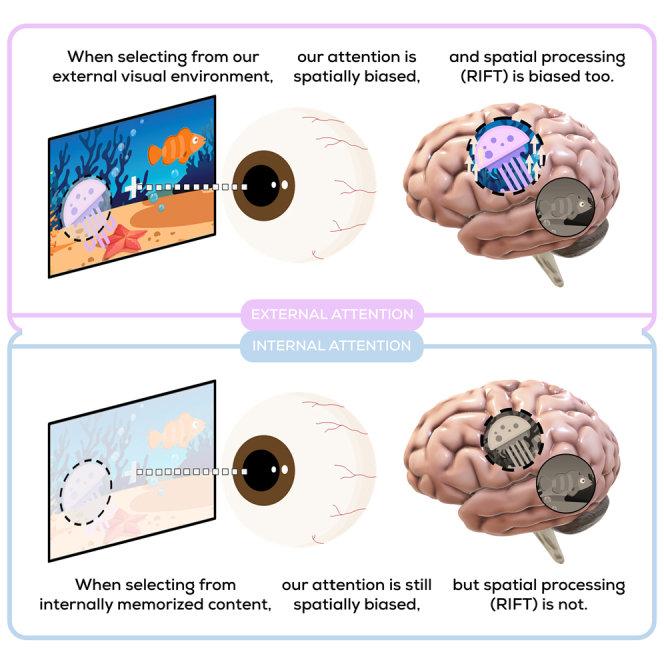

## Introduction

Visual Working Memory (VWM) enables the temporary maintenance of visual stimuli to facilitate perception and upcoming actions. When multiple items are held within VWM, one item might be more relevant than others for an upcoming task. Just as you can shift attention externally to select relevant objects in the visual environment, you can shift attention internally to relevant content in VWM. For example, as you approach the confectionery aisle of the grocery store, you may prioritize the mental representation of your favorite brand of cookies, as opposed to that of milk or bread. Although external attention (prioritizing currently available visual input) and internal attention (prioritizing VWM information that is no longer available externally) are two seemingly different processes, it has been shown that internal attention shares some neural and functional markers of external attention.^1^^,^^2^^,^^3^^,^^4^ Despite such markers of similarity, it is currently unclear to which extent internal and external attentional selection recruit the same neural mechanisms; does our hyper-efficient neural system reflect this similarity by consolidating the mechanisms the two use into one shared pipeline?

The external world is inherently spatially organized; different objects occupy different locations in our environment. During external selection, we may therefore direct our spatial attention to locations with relevant stimuli, thus boosting the processing of visual input at the corresponding locations. Somewhat surprisingly, evidence suggests a similar reliance on location during selection in VWM, even when there is no relevant information at these locations anymore. For example, when asked for the color of your lost phone, you may automatically direct attention to the pocket in which you usually keep it while retrieving its color from memory.

Evidence for a spatial organization in VWM mainly stems from studies using retro-cue paradigms. A retro-cue is presented after a set of items has already been memorized, and informs the participant about the relevance of an item within this set for an upcoming task. Recall performance is better for retro-cued items, suggesting that the cue elevates the item to a prioritized state within VWM.^5^ It has been shown that retroactively cueing an item in VWM produces a behavioral bias toward the location where this item was previously memorized, even if memorizing item locations was not necessary to complete the task. Reaction times to visible targets that are displayed following a retro-cue are faster when they match the location where the retro-cued item was memorized.^6^^,^^7^ In similar tasks, retro-cues induce microsaccadic eye movements toward the previously memorized item location,^8^^,^^9^^,^^10^ as well as desynchronization of alpha power contralateral to the encoding location of the item.^10^^,^^11^^,^^12^^,^^13^^,^^14^ Alpha lateralization is an established neural signature of orienting visual attention.^15^^,^^16^^,^^17^^,^^18^^,^^19^ Taken together, this work shows that even if the former encoding locations of memory items are no longer important (they contain no task-relevant sensory information), maintenance and selection of items in VWM still seem to operate within a spatial layout that mirrors the layout of our external world. Notably, there is an abundance of evidence showing that the visual system is biased toward features of prioritized VWM items.^20^^,^^21^^,^^22^ It is also known that spatial attention is sustained during the maintenance of only one item in VWM.^23^^,^^24^ However, here our focus lies specifically on the spatial biases that emerge during *shifts* of internal attention.

**Figure 1:**
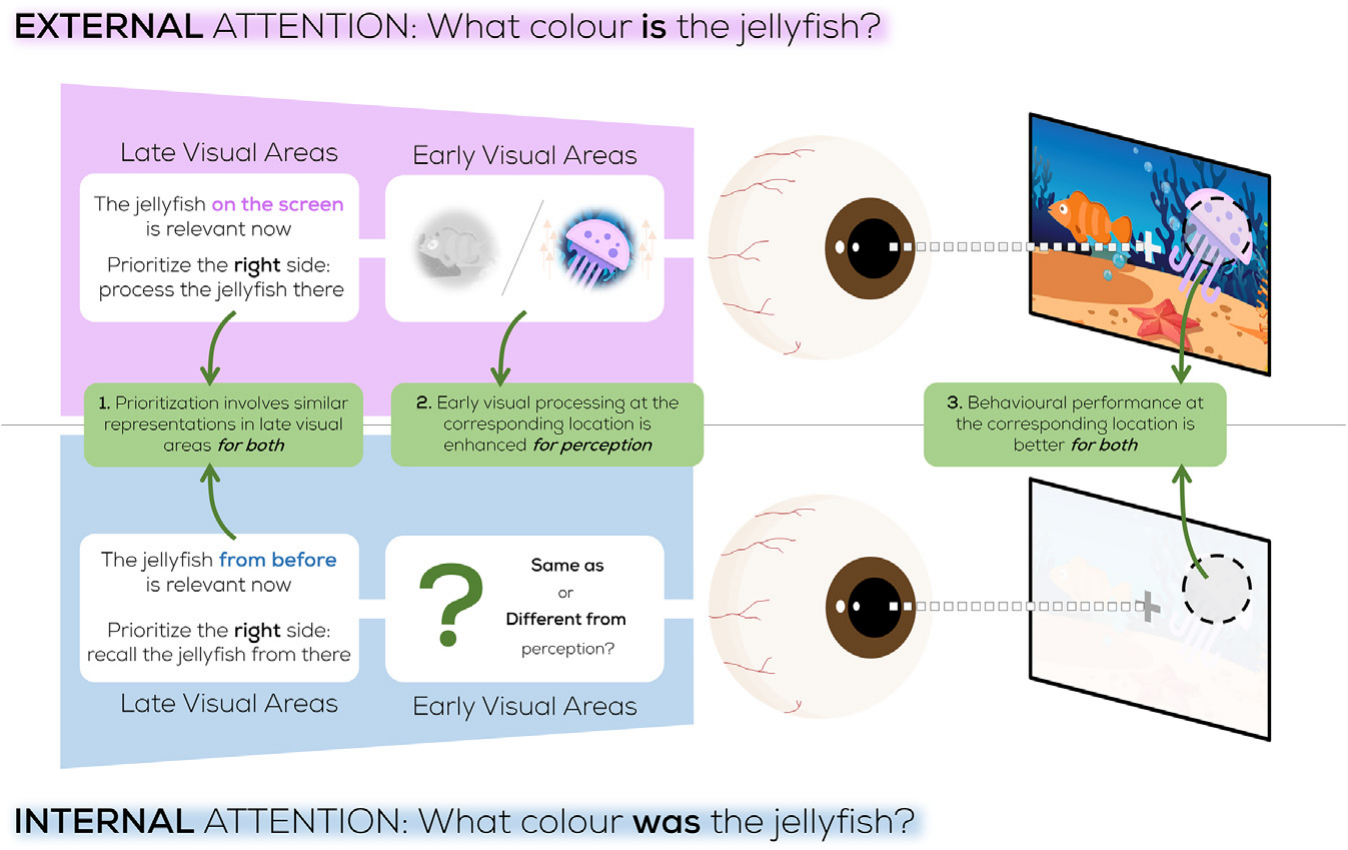
Similarities and differences between internal and external attention Similarities between the representation of location during internal (blue region) and external (purple region) prioritization exist in high-level visual areas (left), and behavioral performance at the corresponding location improves for both (right). It is currently unknown whether early visual processing behaves similarly across both. This may differ because of the different functions involved - encode novel stimuli vs. highlight memorized information. A distinction at the early processing level could indicate whether internal attention is vestigially using spatial mechanisms that evolved for processing the external world, or that this spatial layout within VWM serves a functional purpose.

Despite clear evidence for a spatial organization in VWM, there is one key distinction to be made between such a spatial organization in external and internal attention. Attentional shifts toward (external) perceived stimuli serve the encoding of task-relevant sensory input at that location. In contrast, when a retro-cue instructs participants to favor one stimulus over the other, the former location of the memorized stimulus no longer contains any relevant information. Thus, although the literature reviewed above showed an attentional bias toward the former location of retro-cued stimuli, VWM cannot benefit from this spatial organization in the same way that external attention does. During external attention, excitability of the earliest sensory cortices is boosted at these locations in order to facilitate the encoding of sensory information.^25^^,^^26^ Although internal attention also produces (behavioral and neural) biases toward the attended item location,^7^^,^^8^^,^^10^^,^^11^^,^^12^^,^^13^^,^^14^ this does not automatically imply that these biases are similarly accompanied, or caused, by an enhancement of such early sensory processing. There is support for both the presence and absence of this early sensory enhancement during internal attention. Features of internally prioritized items can be recovered from sensory areas,^27^ suggesting that they are maintained in sensory regions. As these early visual processing regions are inherently retinotopically organized, one might expect that the prioritization of visual memories also leads to a facilitation of the early sensory response to external input at former encoding locations. From a functional point of view, however, such a boost would not serve its traditional advantage of encoding external sensory information, since there is none at these locations. Here, we resolve this intriguing contradiction and test whether internal attention leads to location-specific sensory enhancement just like selecting objects externally (Figure 1).

We want to measure whether sensory processing is boosted at specific locations. This is often studied by presenting a visible target or probe at the corresponding locations. However, this poses two problems: first, the frequent occurrence of probes may lead participants to anticipate the appearance of a visible stimulus, as a result of which participants may allocate spatial attention to the expected probe locations. This makes it difficult to investigate the intrinsic allocation of attention, as would have occurred in the absence of a perceptible stimulus. Second, the processing of a visible probe engages several stages of the visual processing hierarchy. If some attentional manipulation benefits responses to a visible probe, this may be either because attention boosted sensory processing at the probe location (even before the probe was there), or it could be because attention impacted the (downstream) visual response to the probe itself. Though these two possibilities may appear to be inherently indistinguishable, because visual processing is conventionally studied with visible stimuli, our current approach circumvents this problem. We build on recent methodological advances that allow for driving early visual cortex without engaging the downstream visual processing regions commonly associated with conscious perception. Rapid periodic stimulation induces a rhythmic, retinotopic response that predominantly emerges from early sensory areas.^28^^,^^29^ A recent technique known as Rapid Invisible Frequency Tagging (RIFT)^26^ leverages this form of stimulation to offer a measure of early visual processing at specific locations. In doing so, it forms an invisible tracker of spatial attention.

Here, we use RIFT to measure the extent to which early visual processing at locations previously or currently occupied by stimuli is modulated during both internal and external shifts of attention. We show that despite the presence of clear correlates of attention (a lateralization of alpha oscillations and gaze position), early visual processing is not spatially modulated at the previous locations of memorized items (i.e., during internal attention), even though this increase is seen at cued item locations during external attention. We discuss the implications of our results for the mechanism underlying attentional prioritization in VWM, and how they relate to the organizational principles of VWM.

## Results

**Figure 2:**
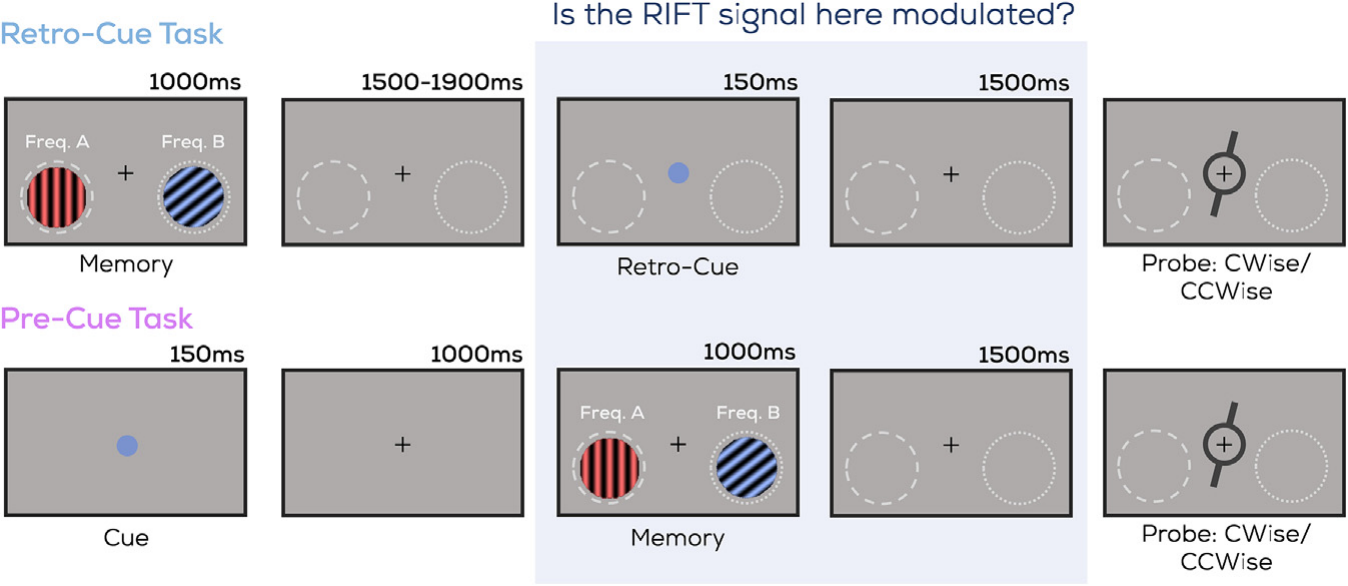
Task design Two uniquely colored and oriented stimuli were presented on either side of fixation. This was followed by (retro-cue experiment) or preceded by (pre-cue experiment) a red or blue circle (the cue), indicating which of the two oriented stimuli (i.e., the red or blue item) would be probed. After a delay, participants indicated whether a memory probe was tilted clockwise or counterclockwise relative to the cued item. The highlighted locations flickered at 60 or 64 Hz (See *Tagging Manipulation*) throughout the trial (Note: not to scale; memory probe did not overlap with flickering regions in actual display. No actual outlines around flickering regions were displayed).

To investigate whether internal attention (prioritizing relevant content in VWM) boosts early visual processing similar to external attention (orienting toward relevant stimuli in the environment), we ran two main experiments each with 24 healthy human participants, differing only in sequence and type of cue. In the retro-cue experiment, participants memorized two items, and following a color cue they made an internal selection within memorized VWM content to report on the retro-cued item’s orientation (Figure 2; top). In the pre-cue experiment, the color cue was displayed first, and during the memory item display participants memorized only one item for report upon selecting it from their external visual environment (Figure 2; bottom). In both experiments, we used RIFT responses to identify spatial biases in visual processing. In an additional control experiment (*n* = 24), discussed further below, we use a spatial cue to confirm the applicability of RIFT to the retro-cue experiment.

Behavioral results from all three experiments showed that participants performed the task successfully. Performance was above chance on the retro-cue experiment (mean = 76.7%, median = 78.3%), pre-cue experiment (mean = 83.4%, median = 85.2%), and spatial-cue experiment (mean = 80.2%, median = 80.5%). All further analysis shown here was conducted using all trials, however, we separately analyzed the data using only correct trials and observed the same results qualitatively.

**Figure 3:**
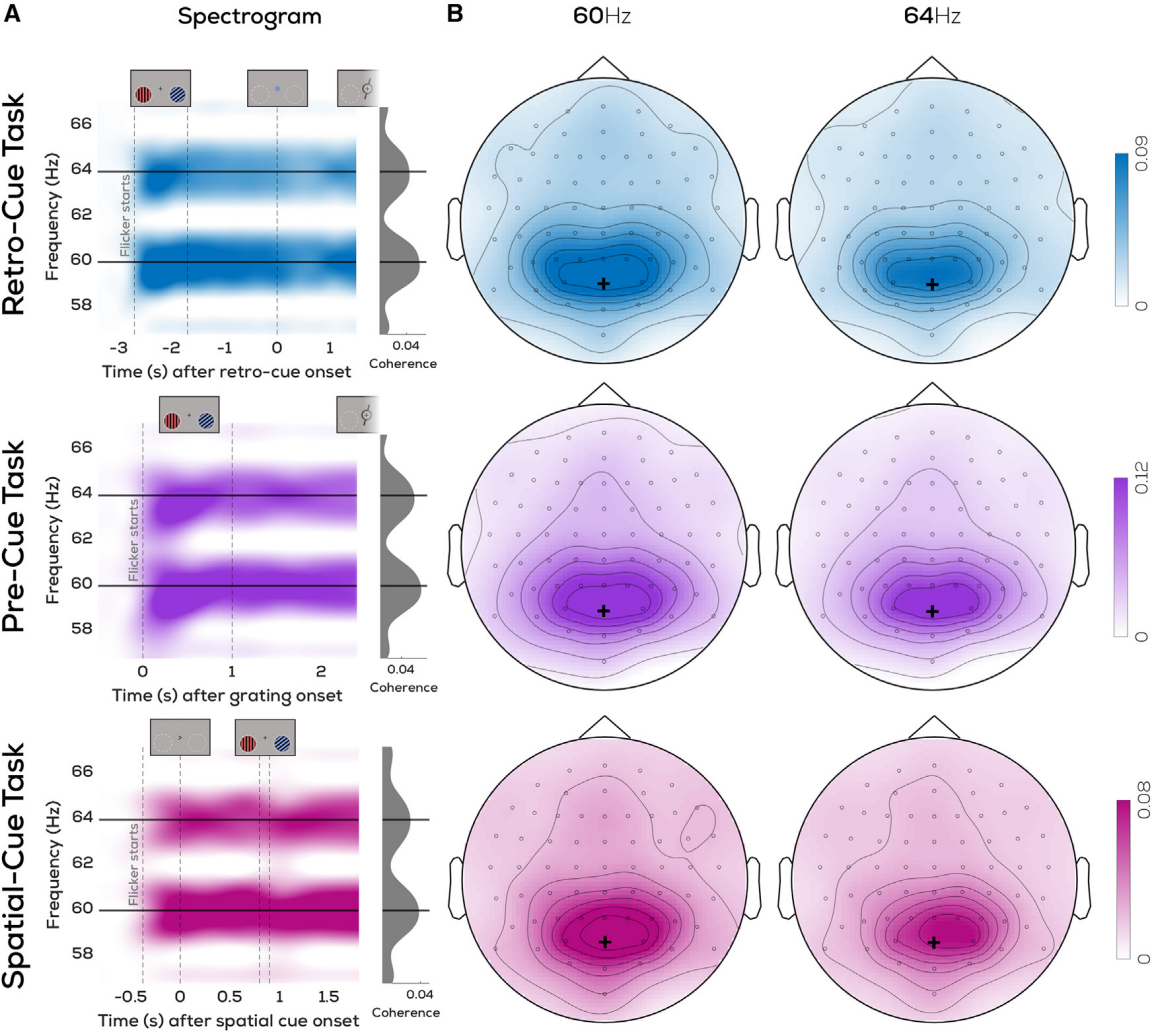
Average RIFT response (A) Time-frequency plot averaged across participants and top 6 channels with highest coherence (selected individually per participant) showing clear peaks at 60 Hz and 64 Hz following flicker onset. (B) Topographical distribution of average RIFT coherence over the interval during which both gratings were on screen. Black plus marks the POz electrode. Note that separate y axis limits are used for the coherence traces and topographies in each experiment, see text under section “*RIFT responses to prioritized locations are enhanced* … ”.

We first show that our tagging manipulation successfully evoked an oscillatory response in the EEG signal as quantified via coherence. Coherence (from individuals’ top 6 channels, see Figure S1 for an overview of selected channels) showed a clear peak at the tagged frequencies (Figure 3A). This was highest around parietal/occipital electrodes (Figure 3B) in both experiments as expected from previous RIFT studies,^28^^,^^29^ showing that we successfully pick up two simultaneous RIFT-tags.

### RIFT responses to prioritized locations are enhanced only during external and not internal shifts of attention

**Figure 4:**
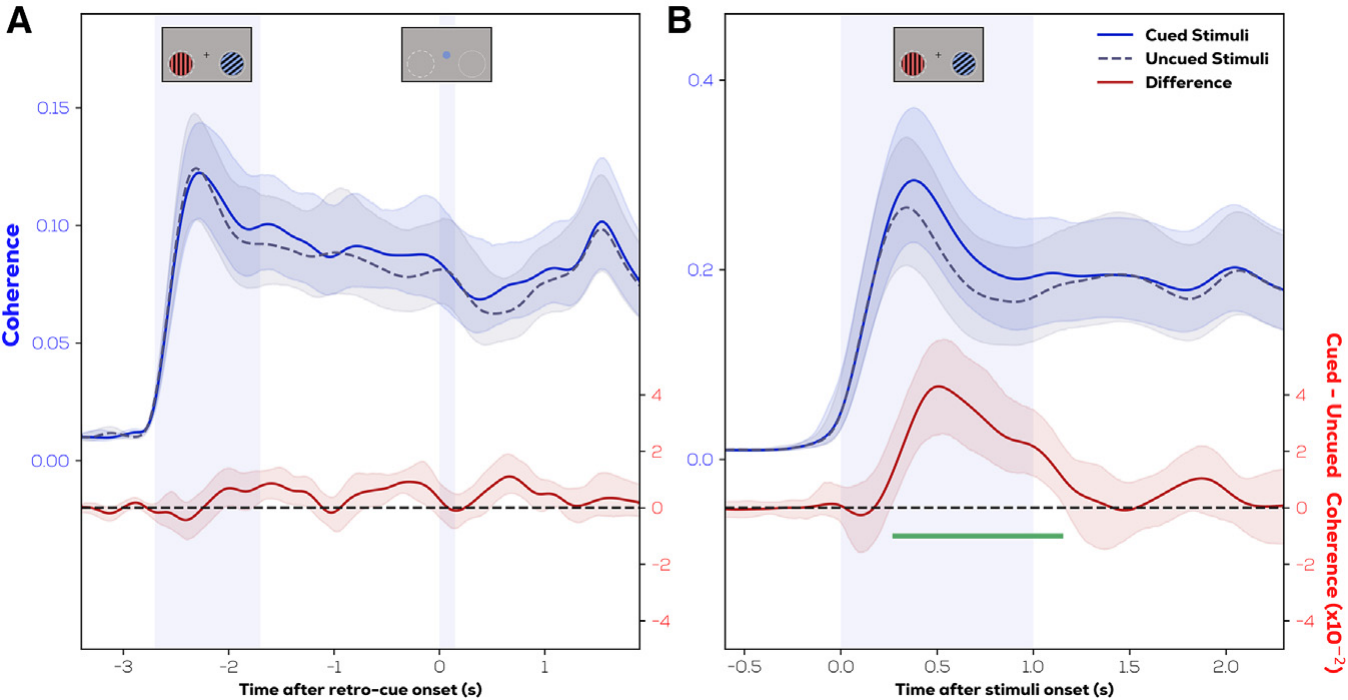
RIFT responses to prioritized locations are enhanced only during external and not internal selection RIFT coherence from frequencies corresponding to cued vs. uncued stimuli (blue) locations and their difference (red) in the (A) retro-cue experiment (B) pre-cue experiment (shaded region—95% bootstrapped CIs). Note that separate y axis limits are used for the coherence traces over both figures to convey equivalent variances visually, see text under section “*RIFT responses to prioritized locations are enhanced* … ”.

We compared the RIFT responses (60 Hz and 64 Hz averaged) elicited from locations of cued and uncued stimuli (Figure 4). In the retro-cue experiment, no difference between the RIFT signal evoked from the cued and uncued item locations was evident following presentation of the retro-cue. However, in the pre-cue experiment, a clear difference in coherence was observed between cued and uncued locations. A cluster permutation test^30^ (green line in Figure 4) showed that in the pre-cue experiment, coherence from cued item locations was higher than that of uncued item locations in the interval from 0.28 s to 1.14 s after stimulus onset (*p* < 0.0005; cluster t-mass compared to permutation distribution). The same test indicated no significant difference between cued and uncued item locations in the retro-cue experiment (*p* = 0.21; largest cluster t-mass compared to permutation distribution). We also replicated these effects for a range of top channel selections (see Figure S2), rather than just the selection of the top 6 channels displayed here. We then compared the attentional modulation between both experiments by averaging coherence within the significant interval of the pre-cue experiment. This was done using a permutation test of mean differences,^31^ which confirmed that the attentional modulation (cued—uncued coherence) in the pre-cue experiment was significantly stronger than that of the retro-cue experiment (mean difference = 0.023, 9% bootstrapped CI of mean differences = [0.013, 0.032], *p* = 0.0002). Here, we average over 60 Hz and 64 Hz, and the same trend is also present for 60 Hz and 64 Hz separately (Figure S3).

Notably, the absolute magnitude of coherence differs considerably between the two experiments, which we believe to be related to technical aspects of the display and EEG sampling, rather than cognitive/neural differences between the two experiments. The variable stimulus-cue onset in the retro-cue experiment allowed for an aliasing effect causing small misalignments (+/− 2 ms) between trials, which in turn slightly decreased the amount of inter-trial phase-locking and resulted in a lower coherence. We confirm later in our linear mixed-effects model (LMM) analyses that this does not cause the difference between experiments described here. We also show later in a third experiment that it is possible to observe an attentional modulation despite this variable onset and reduced coherence. In summary, we find enhanced processing at cued item locations during external shifts of attention but not during internal shifts of attention.

### Alpha oscillations reflect the location (left/right) of the prioritized item during both internal and external selection

**Figure 5:**
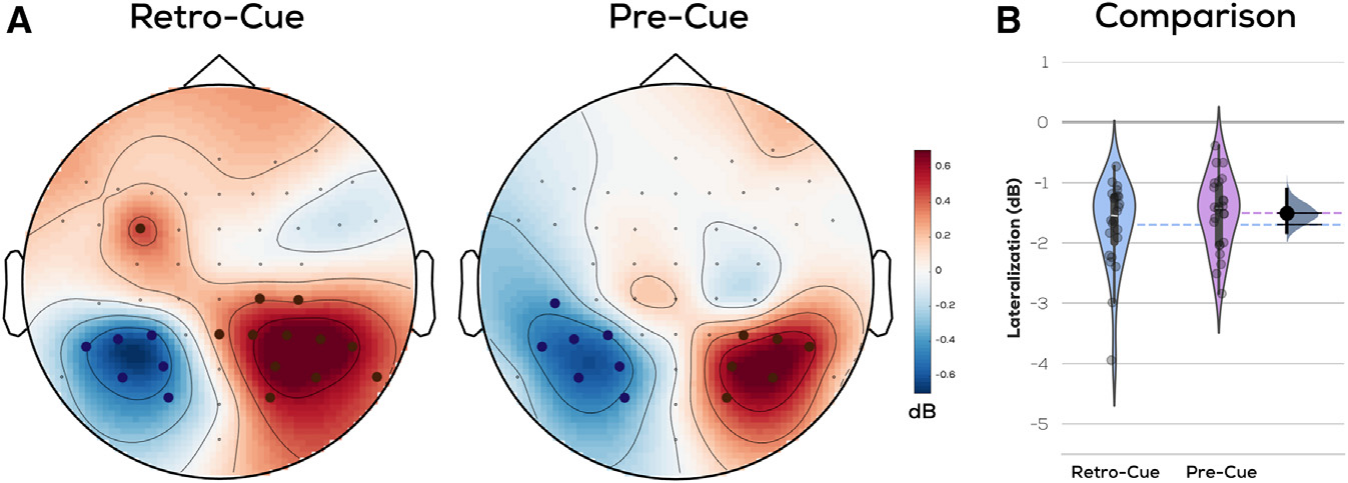
Alpha oscillations reflect the location (left/right) of the prioritized item during both internal and external selection (A) Average difference between alpha power (8–13.5 Hz) in left and right item cued trials upon presentation of retro-cue/stimuli, respectively (channel dots indicate 95% bootstrapped CIs excluding 0, positive = red or negative = blue), (B) Comparison across experiments. Shaded patch indicates kernel density estimation of respective scatterplots; dashed lines indicate respective means; gray indicates distribution of bootstrapped mean differences; black bar indicates 95% Cis.

We next investigated potential lateralizations of alpha oscillations (8–13.5 Hz) in both experiments. Desynchronization of alpha power contralateral to the previous/current location of the cued item has been identified as a marker of spatial attention.^10^^,^^11^^,^^12^^,^^13^^,^^14^^,^^18^ Both the retro-cue and pre-cue experiments showed posterior decreases in alpha power upon presentation of the retro-cue or stimuli, respectively, compared to the baseline (Figure S4). The electrodes showing the strongest decrease in alpha power were contralateral to the direction of the cued item, confirming that both experiments showed strong alpha lateralization (Figure 5A) (retro-cue experiment: mean = −1.70, 95% bootstrapped CI = [−2.06, −1.48]; pre-cue experiment: mean = −1.51, 95% bootstrapped CI = [−1.77, −1.29]; averaged values from significant channels as marked in Figure 5A). We compared alpha lateralization between the two experiments using a permutation test of mean differences,^31^ which revealed an equal degree of lateralization (Figure 5B) across both experiments (mean difference = 0.193, 95% bootstrapped CIs of mean differences = [−0.14, 0.56], *p* = 0.32). This indicates that the previous location of the memorized item, although irrelevant for the task, was indexed by alpha oscillations, similar to what was observed during external attention. Endogenous oscillations therefore suggest that internal and external selection operate with a similar spatial layout. This confirms that people shifted attention to the prioritized item in both our experiments and reflects earlier work suggesting that alpha oscillations index spatial attentional shifts.^15^^,^^16^^,^^17^^,^^18^

### Gaze position also reflects the location (left/right) of the prioritized item during both internal and external selection

**Figure 6:**
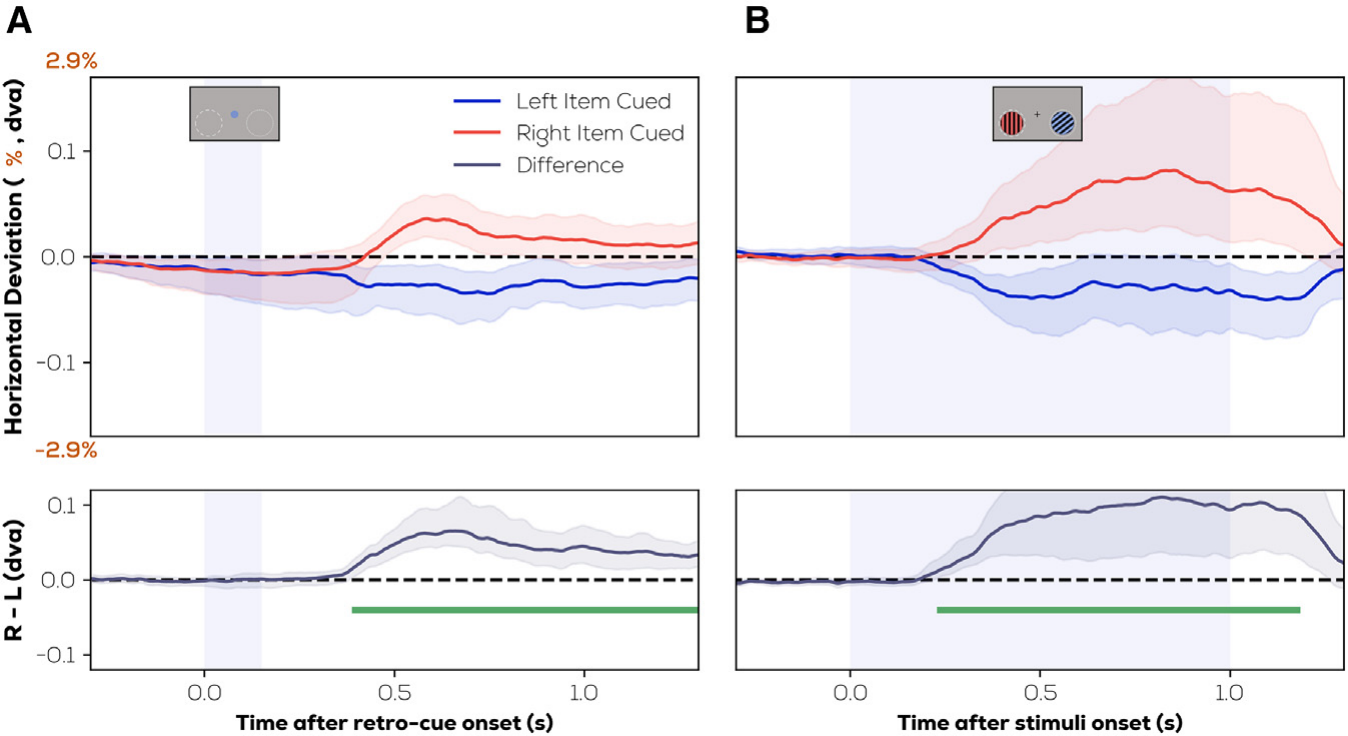
Gaze position also reflects the location (left/right) of the prioritized item during both internal and external selection Difference in gaze position between right cued and left cued trials (top) and difference (bottom) in the (A) retro-cue experiment (B) pre-cue experiment (shaded region—95% bootstrapped CIs). Deviation is reported in terms of dva and percentage of distance from fixation to horizontal eccentricity of stimulus center.

We also looked at shifts in gaze position during both experiments (Figure 6), as these have been identified as correlates of prioritization in VWM.^8^ Participants were instructed to maintain fixation throughout, and any trials where saccades were made to the item locations were excluded. Nonetheless, small deviations in gaze position were observed in the direction of the previous display location of the retro-cued item (*p* < 0.003; cluster t-mass compared to permutation distribution) in the retro-cue experiment (Figure 6A), and of the cued item (*p* < 0.0005; cluster t-mass compared to permutation distribution) in the pre-cue experiment (Figure 6B) using a cluster permutation test^30^ (green line in Figure 6). We also compared the gaze bias between both experiments by averaging it across time within the significant intervals. This was done using a permutation test of mean differences,^31^ which did not show any difference between the gaze biases between both experiments (mean difference = 0.043, 95% bootstrapped CI of mean differences = [−0.011, 0.125], *p* = 0.25). Thus, supplementing the results seen with alpha lateralization, small deviations in gaze position indexed the location where the cued item was previously memorized (retro-cue experiment) or shown (pre-cue experiment), despite this location being irrelevant for the retro-cue experiment.

Collectively, the alpha lateralization and the gaze bias results show substantial evidence of an attentional bias toward the location of the cued item, even in the retro-cue experiment when the memory items were no longer present.

### Gaze position bias does not drive other metrics of attentional modulation

**Figure 7:**
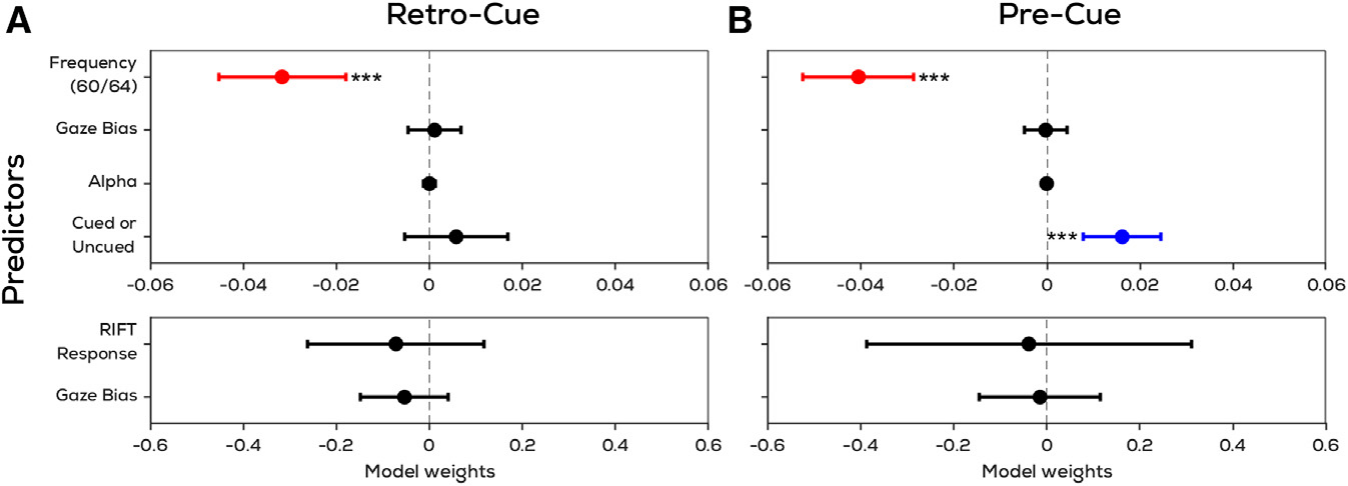
Gaze position bias does not drive other metrics of attentional modulation Linear mixed effects model predicting top: the RIFT response and bottom: alpha lateralization in the (A) retro-cue experiment (B) pre-cue experiment (shaded region—95% bootstrapped CIs). Linear coefficients (β) are reported with error bars representing 95% confidence intervals. The trial-wise gaze position bias does not explain the trial-wise attentional modulation in the RIFT response or alpha lateralization.

The previous results showcase three metrics (RIFT, lateralization of alpha power, and gaze bias) of attention. We ran linear mixed-effects models to investigate to what extent these metrics co-vary, and reflect similar or distinct cognitive mechanisms. We show that in both the retro-cue experiment where the RIFT response was not modulated by attention, as well as in the pre-cue experiment where it was, the trial-wise alpha lateralization and gaze biases did not predict the trial-wise RIFT response (Figure 7; top). This was the case despite accounting for variability across participants and the two frequencies used. The RIFT response is, however, inversely related to frequency, which reflects the fact that 64 Hz tags evoke a weaker response than 60 Hz tags. In the pre-cue experiment the RIFT response is significantly higher from cued than uncued item locations. This reflects the main finding shown earlier with coherence. It additionally eliminates the possibility that the retro-cue experiment having a lower coherence than the pre-cue experiment prevented an attentional modulation from emerging in the former, since the trial-wise Hilbert magnitudes used for the LMM were equivalent between the two experiments (Figure S5) and replicated this result. A second LMM showed that alpha lateralization is also not explained by either of the other two metrics in both experiments (Figure 7; bottom). This suggests that the three metrics reported here (namely RIFT, lateralization of alpha power, and gaze bias) capture distinct cognitive mechanisms.

### Control experiment confirms that attentional modulations of the RIFT response can also be measured in the absence of visible stimuli

**Figure 8:**
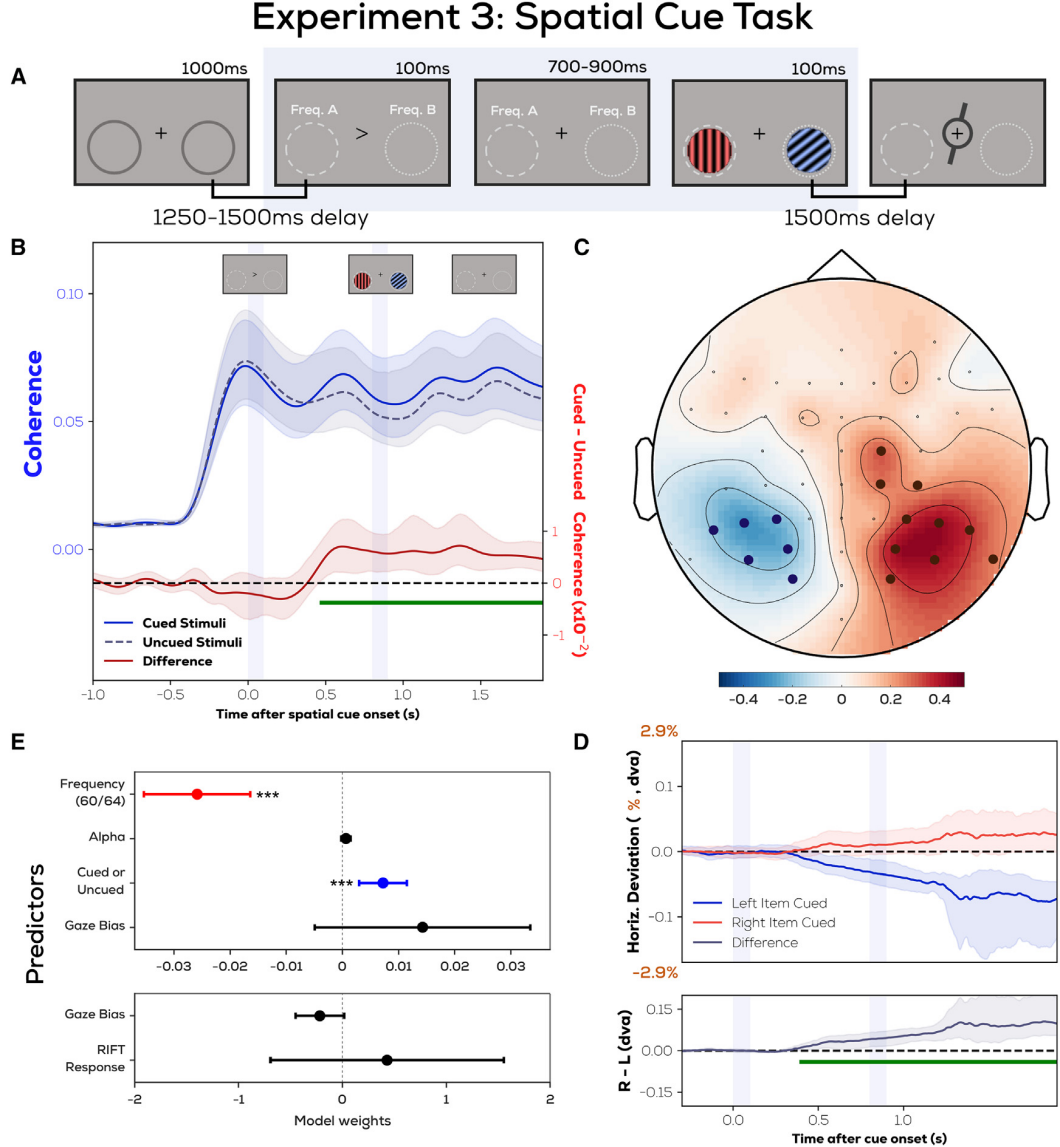
Control experiment with a spatial cue confirms that attentional modulations of the RIFT response can also be measured in the absence of visible stimuli (A) In a spatial-cue experiment similar to the earlier pre-cue experiment, we allowed participants to shift attention before the items were displayed to confirm that RIFT modulation could be observed in the absence of stimuli. (B) The enhanced RIFT response from the cued location was still present both before and after the items were displayed. We also replicated the effects of (C) alpha lateralization and (D) gaze position bias toward the cued location as seen earlier in the retro-cue and pre-cue experiments. (E) Lastly, with Linear mixed effects modeling we (1) confirmed that for this experiment as well gaze position was not driving the RIFT response, and (2) replicated the attentional modulation effect of coherence using trial-wise Hilbert-magnitudes.

A key difference between the retro-cue and pre-cue experiments is that in the duration of interest, the pre-cue experiment had stimuli (gratings) on the screen in the tagged locations. In the retro-cue experiment these locations were tagged, but no perceptual input was present at these locations, since the tagged region appeared invisible against the background. This leaves open the possibility that visual processing is in fact spatially modulated upon presenting the retro-cue, but RIFT is unable to measure this bias unless an actual stimulus is present at the tagged area. To eliminate this possibility, we confirmed that RIFT can indeed measure attentional modulations even to background locations in an additional experiment using a spatial pre-cue (Figure 8A).

Following a spatial pre-cue toward an upcoming item to be memorized, we measure a higher RIFT response from the tagged area in the cued direction, even before any stimuli are displayed. A cluster permutation test^30^ (green line in Figure 8B) showed that coherence from upcoming cued item locations was higher than that of the uncued item locations in the interval from 0.47 s onwards after cue onset (*p* < 0.0005; cluster t-mass compared to permutation distribution). This is earlier than the average stimulus onset (0.8 s after cue onset), as well as the earliest stimulus onset (0.7 s after cue onset). We also compared this spatial difference explicitly between selected time intervals and showed that it is unchanged if we compare time periods before, during, and after the stimuli were on the screen (Figure S6). We also replicated two effects shown earlier for the retro-cue and pre-cue experiments; alpha power was reduced contralateral to the cued side following cue presentation (Figure 8C, average desynchronization difference between right-cued and left-cued trials from 0.4 to 0.7s after cue onset), and gaze position was biased toward the cued location (Figure 8D) from 0.40s onwards after cue onset as shown by a cluster permutation test^30^ (*p* < 0.0005; cluster t-mass compared to permutation distribution). Lastly, linear mixed modeling analysis (Figure 8E) replicated the results seen for the pre-cue experiment, reiterating that the above described metrics of attentional shifts did not correlate. In Figure 8E the trialwise RIFT response (Hilbert magnitude) was averaged across the significant interval in the coherence comparison, but the same results qualitatively were obtained when averaging only the RIFT response before the gratings were on screen or only the RIFT response after the gratings had disappeared. Thus, the spatial-cue experiment confirmed that the difference between the retro-cue and pre-cue experiments was not caused by a difference in sensory stimulation.

## Discussion

We investigated whether internal attention (orienting toward relevant content in VWM) boosts early visual processing at the attended item’s previous location, as is the case for external attention (orienting toward relevant stimuli in the environment). Despite a shift of attention toward the location of the prioritized items during both external and internal selection (as reflected by alpha oscillations and gaze position), early visual processing at locations of attended items is enhanced only during external attention. We thus show that the spatial organization within VWM is not a passive, vestigially derived consequence of internal and external attention utilizing the same neural machinery—which would have biased early visual processing in the same manner across the two. Our results instead suggest that this spatial organization uniquely facilitates their separate functions, and that the visual system uses space as an organizational principle to store and select items in VWM.

Our results are in line with other work providing evidence for a spatial organization of VWM. An established neural signature of attentional prioritization within VWM is the decreased amplitude of alpha oscillations observed in the hemisphere contralateral to the cued item, which we reproduced in both experiments.^10^^,^^11^^,^^12^^,^^13^^,^^14^ This lateralization indicates a spatial layout within VWM since it reflects the location where a prioritized item was previously memorized in the brain even when location is not required for the task. Furthermore, we show that participants’ gaze is biased toward the (now empty) location of the selected item, replicating existing work.^8^^,^^9^ The previous results confirm that internal attention utilizes a spatial reference frame similar to that seen in external attention. Importantly, however, this shared spatial bias does not extend to early sensory processing at the corresponding locations; although we observe a robust boost in sensory processing at cued item locations during external attentional selection, we do not observe this during prioritization in VWM. That is, though a substantial RIFT signal is evoked from the previous locations of prioritized and unprioritized items, these signals are not modulated by the retro-cue.

Behavioral work has shown that responses to visible targets are faster when presented at former locations of prioritized memory items.^7^ This is seemingly in contrast with our results, since we show that early visual processing at these locations is not enhanced. However, responding faster to a visible target at a particular location does not guarantee that visual processing was already enhanced at that location before this stimulus was there. It may simply mean that once a stimulus is presented at that location, this stimulus draws attention faster than it would have elsewhere. Alternatively, participants may expect the appearance of a visible target and strategically allocate external spatial attention to the expected stimulus location in order to encode the target. In contrast to previous work,^7^ our “attentional probes” are invisible, allowing us to measure how spatial attention boosts visual processing at a specific location (without the confound of visible stimuli that alter the allocation of attention). Thus, the behavioral evidence measures faster responses to displayed targets, whereas here we measure whether visual processing is automatically boosted at these locations even in the absence of any such targets. Here, we show that in the case of internal attention, the attended location in space does not evoke enhanced sensory processing. This follows logically from the fact that there is no task-relevant sensory information to process at the attended location. Then, once a stimulus is shown at the corresponding location during the period of prioritization, as is the scenario found within the behavioral evidence,^7^ its processing could then benefit from the attentional bias that already exists more downstream along the visual hierarchy as captured by alpha lateralization.^10^^,^^11^^,^^12^^,^^13^^,^^14^ RIFT predominantly engages low level visual areas which would, in this case, not be engaged during VWM prioritization. There is evidence for this account: a bias in processing of the attended locations can be identified in a similar study using stimuli with visible, low-frequency flickers,^32^ and the previous location of the prioritized item can be decoded in early visual cortex when a visible outline is presented there.^33^ Additionally, during spatial working memory, perceptual input traverses the visual system (in monkeys) faster when originating at the memorized location as compared to others.^34^

The overlap in areas utilized by external and internal processing is discussed within the sensory recruitment hypothesis which posits that VWM storage and perception rely on equivalent patterns of neural activity in the visual cortex.^35^^,^^36^^,^^37^^,^^38^ There is also evidence that features of prioritized VWM items are represented in sensory areas more strongly than those of unprioritized items,^27^^,^^39^ suggesting that neural populations representing prioritized VWM items and externally prioritized items overlap.^40^ It may be inferred from such accounts that internal and external attention relies on the same mechanisms. How does this fit with the lack of a prioritization reflected here in the RIFT response? The two may be independent: shared patterns of activity between internally and externally prioritized items in sensory cortices need not imply that both internal and external attentions are accompanied by a spatially selective boost in visual processing. If VWM representations stored in sensory areas are accessed in a top-down manner, this does not necessarily entail a boosting of concurrent sensory bottom-up input. For example, recently it has been suggested that different cortical layers might be responsible for implementing mechanisms of VWM and perception, respectively.^41^ Thus, our results are not in conflict with existing accounts of similarities between external and internal attention in sensory cortices; they simply show that although the neural mechanisms for representing visual contents during VWM and perception may be shared (i.e., sensory recruitment), the mechanisms for executive processes during VWM and perception (e.g., attentional selection) are not.

In retro-cue experiments such as ours, the location of encoding is not relevant throughout the task. What use is there then in having this spatial organization in VWM at all? The visual system is constantly processing the world in a spatial manner; it is fine-tuned to parse items across some coordinate or location system. Therefore, whether necessary or not, it may be easiest to organize, store, and retrieve VWM content with respect to this spatial coordinate system. This is akin to the “method of loci”, where items are supposedly better-remembered when mentally assigned to unique locations, or spatial contexts, which results in better memorization both in memory athletes as well as in naive participants upon training.^42^^,^^43^ There is support for such a spatial layout from evidence that locations (as compared to other visual features) play a special role in maintaining VWM; encoding VWM items at the same location instead of at unique locations decreases performance,^44^^,^^45^ feature bindings are stronger when locations are not shuffled,^46^ VWM performance is better for stimuli memorized on different depth planes rather than on the same depth plane,^47^ and the availability of location information before report improves memory performance more than the availability of orientation^48^ or color^49^ information. Thus, location has a special role among the features of content in VWM, and a spatial coordinate system is employed to better organize and recall memorized items.

Here, we refer to “early visual processing” as the retinotopic response in visual cortex to contrast changes in the environment, which is captured by our RIFT measure. Given the novelty of this key method used in this study, it is first worth reflecting on this connection and on what the RIFT response actually represents. RIFT gives us the response to rapidly flickering visual stimuli, the luminance of which changes too fast to be perceived but not too fast to evoke a neural response. We thus use RIFT to measure the extent to which the visual cortex modulates its response to (imperceptible) contrast changes at these locations. It has been shown using source localization in MEG studies that the RIFT response predominantly reflects the earliest stages of visual processing at the level of V1,^28^^,^^29^ and that the topographic spread of brain areas responding to RIFT is a smaller and earlier subset of that forming the source of, for example, alpha oscillations.^29^ With this evidence on the RIFT response being localized specifically to early visual processing, our results are therefore not in conflict with previous work suggesting commonalities between external and internal attention more upstream in the visual hierarchy, such as the retro-cue/stimuli evoked lateralization of alpha oscillations that we also observe here.

Since this is among the first studies to combine RIFT with EEG,^50^^,^^51^ the question may arise of whether we simply lack sensitivity to pick up attentional effects in the retro-cue experiment. In our spatial-cue experiment, we show that it is possible to pick up an attentional RIFT modulation even when there is nothing visible displayed on the screen (as is the case for the retro-cue experiment), and with a coherence amplitude even lower than that of the retro-cue experiment. We also show that attention does modulate the RIFT signal in our pre-cue experiment, and all three experiments maintained identical parameters in terms of the eccentricity and size of the flickering region. These factors, combined with previous work where invisible background-flickers have been shown to evoke neural responses modulated by attention,^52^ indicate that the method implemented here is sensitive enough to pick up on covert shifts of attention in the retro-cue experiment.

We also eliminated the possibility that the attentional RIFT modulation was actually caused by eye movements (which could bring the attended stimulus closer to the fovea, thereby enhancing the RIFT response), using linear mixed-effects analysis. Additionally, if RIFT modulations were explained by gaze position alone, we would also see an attentional modulation in RIFT for the retro-cue experiment where a gaze position bias is indeed visible. Notably, eye movements also did not predict alpha lateralization as shown previously.^14^

Finally, we also show that the two neural markers of attention observed during shifts of external attention—namely alpha lateralization and the attentional RIFT modulation—do not co-vary. This is in agreement with previous work on alpha oscillations and frequency tagging.^53^^,^^54^ It has been suggested that modulations in alpha oscillations reflect a gating of visual information to downstream regions of the visual system.^55^ This explains our observations: if the role of alpha oscillations is not to modulate excitability at the early sensory level, but rather to control the flow of information to regions further downstream in the hierarchy, it is not surprising that alpha lateralizations are observed here in the retro-cue experiment despite the absence of RIFT modulations. In the context of VWM, alpha oscillations are known to be load-dependent,^56^^,^^57^^,^^58^ reflecting an inhibition of visual input to better preserve memory content.^59^^,^^60^ Alpha lateralizations seen during internal attention may imply that preserving prioritized items in VWM requires alpha oscillations to control the spatial distribution of processing in downstream visual regions, without needing to spatially modulate the low-level sensory response. Thus, during prioritization in VWM, location is reflected at a level that includes alpha oscillatory activity, perhaps involving our internal representations of memorized objects, but location is not reflected at a level that includes early visual processing. That is, internal attention does not alter the extent to which the visual cortex is responsive to contrast changes at previous memory locations.

### Conclusion

Here, we investigated the role of early visual processing during attentional selection from perception and from VWM. We show that while both types of attentional selection operate within a spatial layout that reflects the spatial organization of our sensory environment, only external attention modulates visual processing at specific locations. If a sensory boost was observed during internal attention, then its spatial layout may have been explained by internal attention vestigially displaying features of a mechanism that evolved for attending in the external world. By revealing a distinction between the mechanisms underlying internal and external attentional selection, our results show that internal attention is instead using location as an organizational principle to store and select items in VWM. We also show that although the neural mechanisms for representing visual contents during VWM and perception may be shared (i.e., sensory recruitment), the mechanisms for executive processes during VWM and perception (e.g., attentional selection) are not.

### Limitations of the study

Here, we compare biases in spatial attention during internal and external prioritization. A key factor in this comparison is that the RIFT stimulus we use here does not propagate beyond early visual cortex, i.e., that it is “invisible” and not perceived by the participant. Although its cortical spread has been localized with MEG work,^28^^,^^29^ recent research has validated its imperceptibility experimentally,^61^ and questionnaires used in this study reiterate this invisibility to our participants, we are unable to directly measure whether the RIFT signal here arises from a particular section of visual cortex given the spatial resolution offered by EEG.

## Resource availability

### Lead contact

Requests for further information and resources should be directed to and will be fulfilled by the lead contact, Kabir Arora (k.arora@uu.nl).

### Materials availability

Not applicable; no specific reagents, cell cultures, or animal models were used for this study.

### Data and code availability

Raw and processed EEG/Eyetracking data, along with code corresponding to analysis and figures has been deposited in OSF and are publicly available as of the date of publication at https://doi.org/10.17605/OSF.IO/YRH6V.

## Acknowledgments

This project receives funding from the 10.13039/501100000781European Research Council (ERC) under the European Union’s Horizon 2020 research and innovation programme (grant agreement n° 863732). We would like to thank Freek van Ede for assistance in analyzing our eye movement data. We would also like to thank Laura van Zantwijk for assistance with data collection.

## Author contributions

Conceptualization, K.A., S.C., S.G., S.V.d.S., and L.K.; data curation, K.A.; formal analysis, K.A. and S.C.; funding acquisition, S.V.d.S.; investigation, K.A.; methodology, K.A., S.C., and S.G.; project administration, K.A. and S.C.; resources, S.V.d.S.; software, K.A. and S.C.; supervision, S.C., S.G., S.V.d.S., and L.K.; visualization, K.A.; writing—original draft preparation, K.A., S.C., and S.G.; writing—review and editing, K.A., S.C., S.G., S.V.d.S., and L.K.

## Declaration of interests

The authors declare no competing interests.

## STAR★Methods

### Key resources table

**Table:** 

REAGENT or RESOURCE	SOURCE	IDENTIFIER
**Deposited data**		

New Data	https://osf.io/yrh6v/	

### Experimental model and study participant details

We recruited a total of 72 healthy participants (24 each for the retro-cue, pre-cue, and spatial-cue experiments; age: 22.58 ± 2.49 years old, mean +/− std; sex assigned at birth: 52 female, 20 male) with normal or corrected-to-normal vision. None of the participants reported a history of epilepsy or psychiatric diagnosis. Participants provided informed consent at the beginning of the session, and were compensated either with €20 or an equivalent amount of participation credits as per Utrecht University’s internal participation framework. The study was carried out in accordance with the protocol approved by the Faculty of Social and behavioral Sciences Ethics Committee of Utrecht University.

### Method details

#### Protocol

Participants underwent a 2-h experimental session at the Division of Experimental Psychology, Utrecht University. Participants received procedural information prior to the session, and provided informed consent, date of birth, assigned sex at birth, and dominant hand information at the beginning of the session. After completion of the EEG setup, participants were seated 76cm from the screen with a chinrest. After eye-tracker calibration, the experiment was explained using a visual guide and verbal script. Participants of the retro-cue and pre-cue experiments were also informed that they may potentially see visual glitches or flickers on the screen (due to the high-speed projection and tagging, see *Tagging Manipulation*), and that they would be asked after the experiment whether they did or not. Following these instructions and 10 practice trials, the ∼1 h experiment was completed. Participants of the retro-cue and pre-cue experiments filled out a questionnaire on whether they noticed any visual artifacts on screen (and if so, at what stage of the task, and to what degree they felt this interfered with their task on a scale of 1–5), which confirmed that tagging was difficult to perceive (see *Tagging Manipulation*). Compensation was awarded when applicable, and the session was ended.

#### Experimental design and procedure

Two oriented and uniquely colored (blue or red) gratings were presented on both sides of fixation, and participants performed a delayed orientation change detection task on one of them as informed by a color cue. There were three different experiments; one in which the cue was presented after the gratings (retro-cue experiment; Figure 2; top), one in which a color cue was presented prior to the gratings (pre-cue experiment; Figure 2; bottom), and one in which a spatial cue was presented prior to the gratings (spatial-cue experiment; Figure 8).

#### Encoding and cue

##### Retro-cue experiment

Trials began with a centrally presented fixation cross (uniformly random duration between 1 and 1.25s), after which both memory items (gratings) were presented (1s). This was followed by a variable delay (uniformly random, 1.5–1.9s) after which a central retro-cue was presented (0.15s) in the form of either a blue or red circle, informing participants which item would be probed on that trial.

##### Pre-cue experiment

Trials began with a centrally presented fixation cross (uniformly random duration between 1 and 1.25s), after which a central cue was presented (0.15s) in the form of either a blue or red circle, informing participants which upcoming item would be probed on that trial. This was followed by a fixed delay (1s), at the end of which both memory items were presented (1s).

##### Control (spatial-cue) experiment

Trials began with a centrally presented fixation cross and two circular outlines which indicated the location of the upcoming stimuli (1s). Then, after a delay (uniformly random duration between 1.25s and 1.5s), a central spatial cue was presented (0.1s) in the form of an arrow pointing left or right, informing participants which upcoming item would be probed on that trial. This was followed by a variable delay (0.7–0.9s), at the end of which both memory items were presented (0.1s).

Beyond this crucial difference in the order of cue-grating presentations, or the type of cue, the two main experiments as well as the control were identical in the stimuli used.

#### Report

After a fixed cue-probe or grating-probe interval (1.5s), the memory probe was displayed centrally until response. Participants had to specify with a keyboard button press whether the orientation of the memory probe was more clockwise (Q key) or anti-clockwise (P key) compared to that of the cued memory item. Participants were able to respond no sooner than 0.5s after the memory probe onset (indicated via a change in the color of the fixation cross from black to green). Lastly, feedback in the form of a green check mark or red cross was given for correct or incorrect trials respectively. If no response was received within 3s, or a different button was pressed, the trial ended (median = 0.20% of trials, mean = 0.31% of trials).

Each participant completed 480 trials (excluding 10 practice trials, not included in analysis) divided into 15 blocks of 32 trials each. The retro-cue/pre-cue that was blue or red instructed participants to compare the probe orientation to either the orientation of the blue or the red memory item respectively. Orientations of the memory items (always distinct), as well as whether the blue/red stimuli would be presented to the left or right, were equated in prevalence separately and presented in random order. The location of the tagging frequencies (60L/64R vs. 64/60R) and the direction (left/right) of the cued item were counterbalanced and presented in a random sequence.

#### Stimuli

The screen background was maintained at gray (grayscale: 127.5) throughout the experiment. A black fixation cross (0.4 degrees of visual angle; dva) was present in the center of the screen throughout each trial. Memory stimuli consisted of circular square wave gratings (r = 3 dva, spatial freq. = 2 cpdva) either blue or red in color. These were presented slightly below horizontal (eccentricity = 6 dva horizontal, −2 dva vertical) in order to facilitate the tagging response.^62^ To remove visible edges at the boundary of the tagged region, a radially symmetric transparency mask was applied to the memory stimuli as per Equation 1.

(Equation 1) $$T\left(x\right)=\frac{1}{1+e^{\frac{-6x}{r}}}$$

where *x* is the distance of a point on the circular patch from center (0) to the circumference (at radius *r*), and *T(x)* is the resulting transparency at this point ranging from 0.5 (semi-transparent) to 1 (fully opaque). Both memory stimuli were presented with distinct orientations ranging from 0 to 165° in intervals of 15° (thus covering the full range of possible orientations in discrete steps). The circular outlines presented at the start of the spatial-cue experiment had the same position and radius as the gratings. The cue in both retro-cue and pre-cue experiments (*r* = 0.23 dva) was displayed centrally in one of the two memory stimuli colors. The cue in the spatial-cue experiment consisted of two lines drawn within a central square (side = 0.28 dva), with either the left corners connected to the right-midpoint (right-cued) or the right corners connected to the left-midpoint (left-cued). The memory probe consisted of a black, circular annulus (*r* = 1.2 dva, thickness = 0.13 dva) with spokes (0.6 dva extensions) protruding outwards from diametrically opposite ends to indicate an orientation. These dimensions ensured that the tips of the memory probe never overlap (minimum separation = 1.5 dva) with the flickering regions on screen (See *Tagging Manipulation*). The orientation of the memory probe could vary between 3° and 50° clockwise or anti-clockwise relative to the cued memory stimulus. Its orientation relative to the cued item varied on a trial-by-trial basis, following a staircase procedure (PsychtoolBox QUEST algorithm^63^) that targeted 75% accuracy on the memory task (β = 3.5, Δ = 0.01, γ = 0.5).

#### Tagging Manipulation

We used Rapid Invisible Frequency Tagging (RIFT) to evoke an oscillatory response from specific locations on the screen.^26^^,^^64^ This involved sinusoidally varying the screen luminance at certain locations at specific frequencies. In the retro-cue and pre-cue experiments, the areas corresponding to the two memory items were tagged from grating onset until the end of the trial. In the spatial-cue experiment, tagging begun variably between 0.25 and 0.5s prior to cue onset, and remained until the end of the trial. Two frequencies (60Hz and 64Hz) were randomly assigned to either the left or right area, resulting in two possible configurations: 60 left, 64 right; or 64 left, 60 right. When memory stimuli were presented, the two circular gratings were tagged with the corresponding frequency. For the remainder of the trial, the background at the two stimuli locations was tagged (from white to black in order to look invisible against the gray background). The same transparency map was applied to the flickering regions as the memory stimuli (See *Stimuli*). The tagging sinusoids were phase locked to cue onset in both experiments. Prior to data collection, the displayed tagging frequency was verified using a BioSemi PhotoCell luminance sensor (BioSemi B.V., Amsterdam, The Netherlands). Temporal precision of the displayed stimuli was continually recorded during data collection using PsychToolBox’s Screen(‘Flip’) command. Any trial with a frame displayed >4ms off-time was excluded from analysis (median = 0% of trials, mean = 0.17% trials). Given recent research that has established the subjective undetectability of RIFT,^61^ as well as the fact that out of 48 participants in the retro-cue and pre-cue experiments only 4 reported seeing any abnormality on the screen, of which only 1 reported (low-moderate) distraction from the task, we concluded that our tagging was sufficiently difficult to perceive.

#### Display apparatus

Stimuli were projected using a ProPixx projector (VPixx Technologies Inc., QC Canada; resolution = 960x540px; refresh rate = 480Hz) in a rear-projection format (projected screen size = 48 × 27.2cm). Experimental code was written in MATLAB,^65^ using PsychToolBox3^66^^,^^67^ for task display.

#### EEG recording and pre-processing

EEG data were recorded using a 64-channel ActiveTwo BioSemi system (BioSemi B.V., Amsterdam, The Netherlands) at 2048Hz. Two additional electrodes were placed above and on the outer canthus of the left eye respectively. Immediately prior to the experiment, adequate signal quality from all channels was ensured using BioSemi ActiView software. All data analysis was conducted in MATLAB using the Fieldtrip toolbox.^68^ The EEG data were first re-referenced to the average of all channels (excluding poor channels determined by visual inspection, median = 12 [mainly frontal] channels, mean = 11.8 channels). Data were high-pass filtered (0.01Hz), then line noise and its harmonics were removed using a DFT filter (50, 100, 150Hz). Data were segmented into trials ranging from 3.4s before to 2s after retro-cue onset (retro-cue experiment), 2s before to 2.5s after stimuli onset (pre-cue experiment), or 1.5s before to 2.05s after cue onset (spatial-cue experiment). An ICA was performed to remove oculomotor artifacts, and trials with other motor artifacts were removed from further EEG analysis as per visual inspection (median = 11.4%, mean = 13.6%). Baseline correction was performed by averaging (and then subtracting from the signal) a window 0.8s–0.3s before memory stimuli onset in the retro-cue experiment, 0.8s–0.1s before cue onset in the pre-cue experiment, and 1s–0.5s before cue onset in the spatial-cue experiment. Two participants (from the retro-cue experiment) with excessive noise (>50% trials labeled as artifacts) were excluded from further EEG and eye-tracking analysis.

#### RIFT response: Coherence

In order to determine the strength of the EEG response to RIFT frequencies, magnitude-squared coherence was used, which is a dimensionless quantity (ranging from 0 to 1) that measures how consistently similar two signals are in both their power and phase. This results in higher values when two signals i) oscillate at the same frequency, and ii) maintain the same phase difference across trials (i.e., oscillatory responses across successive trials are consistently phase-locked). Coherence was computed between a reference wave (pure sinusoids with the corresponding frequency of 60Hz or 64Hz, sampled at 2048Hz) and condition-specific sets of trials per channel and participant. Segmented trials were first bandpass filtered (±1.9Hz) at the frequencies of interest (60Hz & 64Hz) using a two-pass Butterworth filter (4th order, hamming taper). The filtered time-series data were Hilbert transformed. This provided a time-varying instantaneous magnitude (*M(t)*) and phase (*φ(t)*). The set of all instantaneous magnitudes of the filtered responses ($\vec{M_{x}}\left(t\right)$) and the reference sinusoid ($\vec{M_{y}}\left(t\right)$) across all *n* trials, as well as the differences between their instantaneous phases across all *n* trials ($\Delta \vec{\phi_{xy}}\left(t\right)$) were used to compute time-varying coherence as per Equation 2:

(Equation 2) $$coh\left(t\right)=\frac{{\left|\sum_{tr=1}^{n}\;\vec{M_{x}}\left(t\right)\;\vec{M_{y}}\left(t\right)\;e^{i\Delta \vec{\phi_{xy}}\left(t\right)}\right|}^{2}}{n\sum_{tr=1}^{n}\;\vec{M_{x}}{\left(t\right)}^{2}\;\vec{M_{y}}{\left(t\right)}^{2}}$$

In order to compute coherence spectrograms, coherence was computed for frequencies ranging from 56.8Hz to 67.2Hz in 0.8Hz intervals. In line with previous similar studies, we identified the top 6 channels per individual^51^^,^^69^ with the strongest coherence at 60/64Hz across all trials, and any further comparisons across experimental conditions presented here were made using traces averaged across these channels. However, to confirm that the results of these comparisons are robust across a range of selected channels, we show in Figure S2 that our main takeaways presented in Figure 4 are unchanged regardless of how many channels we select.

#### Alpha Lateralization

Alpha lateralization is an established neural signature of orienting visual attention. We thus investigated whether alpha (∼10Hz) oscillations decreased contralateral to the hemifield where the cued item was encoded (retro-cue experiment), displayed (pre-cue experiment), or about to be displayed (spatial-cue experiment). Time-Frequency representations were computed using the ft_freqanalysis function in the Fieldtrip toolbox.^68^ First, spectral analysis was performed on individual trials in the 8–13.5 Hz range (in increments of 0.2Hz). This was done for every channel using 3-cycle Morlet wavelets and baselined (dB; 10∗log10(signal/baseline)) with respect to a window 0.6s–0.3s before memory stimuli onset (retro-cue experiment) or before cue onset (pre-cue and spatial-cue experiments). The 8-13.5Hz frequency range was then averaged to reflect alpha power, and used to assess the differences in alpha band activity between conditions for each channel. Lateralization of alpha power was visualized and quantified by subtracting the alpha power of left-cued trials from right-cued ones.

#### Eye-tracking recording and analysis

Gaze was tracked using an Eyelink SR (SR Research, Ontario, Canada) eye-tracker. Both eyes were tracked at 500Hz. Immediately prior to the experiment, a 9-point calibration was performed. This calibration was repeated after every 3rd experimental block.

The data were segmented into trials of −1.5s before to 1.5s after retro-cue onset (retro-cue experiment), stimuli onset (pre-cue experiment), or cue onset (spatial-cue experiment). Blink correction was carried out using custom code adapted from existing work.^70^ Trials were baseline-corrected with respect to the average position in an 800ms window prior to retro-cue, stimuli, or spatial-cue onset (in the retro-cue, pre-cue, and spatial-cue experiments respectively). Trials where fixation was not maintained (defined as gaze being further than 2 dva from fixation for more than 50ms of the trial duration) or trials where the baseline was over 2 standard deviations away from the mean baseline position were removed from further eye-tracking analysis (median = 14.69% of trials, mean = 16.41% of trials). The EEG results presented here do not exclude all these trials, however, we separately ran the EEG analysis without these trials which resulted in the same results qualitatively. Three participants (retro-cue experiment) were excluded from gaze position bias analysis for failing to maintain fixation in more than half of the trials. A 10ms uniform smoothing filter was applied to the individual position data.

### Quantification and statistical analysis

#### General statistical analysis

Differences in time-varying measures (coherence and gaze position) across conditions were compared over the duration of a trial using a non-parametric cluster-based permutation test. Coherence traces were first averaged across the top 6 channels per individual with the strongest RIFT coherence at 60/64Hz. This produced a single coherence trace over time per participant for each condition and each frequency. Similarly, both eyes were averaged to produce a single horizontal bias trace over time per participant per condition. Then, a permutation test^30^ was used to inspect differences across attentional conditions. This consisted of four steps. 1) Two traces being compared were subtracted to produce a difference trace. 2) A one-sample t-test was run for each individual time point to detect points on the difference trace significantly different from 0 (*p* < 0.05). Clusters of consecutively significant timepoints longer than 10ms were identified and their sum of t-values was computed within each cluster to produce a cluster-level t-mass. 3) Then, we randomly flipped the sign of each individual difference trace (hence preserving autocorrelations between timepoints in the null data). We conducted 10,000 repetitions of this process to generate a distribution of expected t-mass values given randomized labels. 4) Finally, we checked whether the t-masses of any initially observed clusters were higher than 95% of this distribution. These clusters were accepted as significant. With coherence, we compared the RIFT response evoked from cued vs. uncued item locations first separately for both frequencies, and later averaged. For gaze, we compared horizontal gaze position for cued left vs. cued right trials.

To compare the degree of attentional modulation in the RIFT response and the lateralization of alpha oscillations between the retro-cue and pre-cue experiments, we used a permutation test of mean differences.^31^ Given two sets of data points (here, one value per participant from the retro-cue and the pre-cue experiments), this test uses bootstrap resampling to draw a distribution of the difference between the means of the two sets. On each of its 5000 repetitions, it samples points (with replacement) from both sets, and computes the difference in the means of these two sample sets. The two sets of data points are accepted as significantly different if the 95% confidence interval of this distribution excludes 0.

#### Linear mixed-effects modeling

We lastly used a Linear Mixed-effects Model (LMM) to assess whether the attentional modulation found in the RIFT response could be explained by the other two effects observed, namely alpha lateralization and the gaze bias toward the cued item. LMM analysis was conducted in MATLAB,^65^ using the fitglme function. The corresponding formula used was RIFT Response = 1 + Cued/Uncued + Frequency (60/64) + Gaze Position Bias + Alpha Lateralization + (1 + <all predictors>|Participant). For each recorded metric, a trial-wise, time-averaged value was obtained by averaging the significant interval at the group level as determined by the cluster-based permutation tests. To obtain trial-wise measures of the RIFT response, we used the trial-wise magnitude resulting from the Hilbert transform of the EEG signal (See *‘RIFT Response: Coherence’*), as opposed to coherence, which is computed across a set of trials. For the RIFT response in the retro-cue experiment, where no attentional modulation was found, we used the significant interval from the RIFT response in the pre-cue experiment. Trial-wise alpha lateralizations were computed using channels significant at the group level, by subtracting the ipsilateral from the contralateral alpha power on each trial. A second LMM assessed whether alpha lateralization could be explained by the other effects. The corresponding formula used was Alpha Lateralization = 1 + RIFT Response + Gaze Position Bias + (1 + <all predictors>|Participant).
