# Supplementary material for: Dissociating external and internal attentional selection
Source: iScience. 2025 Mar 25;28(4):112282. doi: 10.1016/j.isci.2025.112282 (PMC12005331; doi:10.1016/j.isci.2025.112282)
Supplement: Document S1. Figures S1–S6 [file mmc1.pdf]

iScience, Volume *28*

Supplemental information

Dissociating external and internal attentional selection

Kabir Arora, Surya Gayet, J. Leon Kenemans, Stefan Van der Stigchel, and Samson Chota

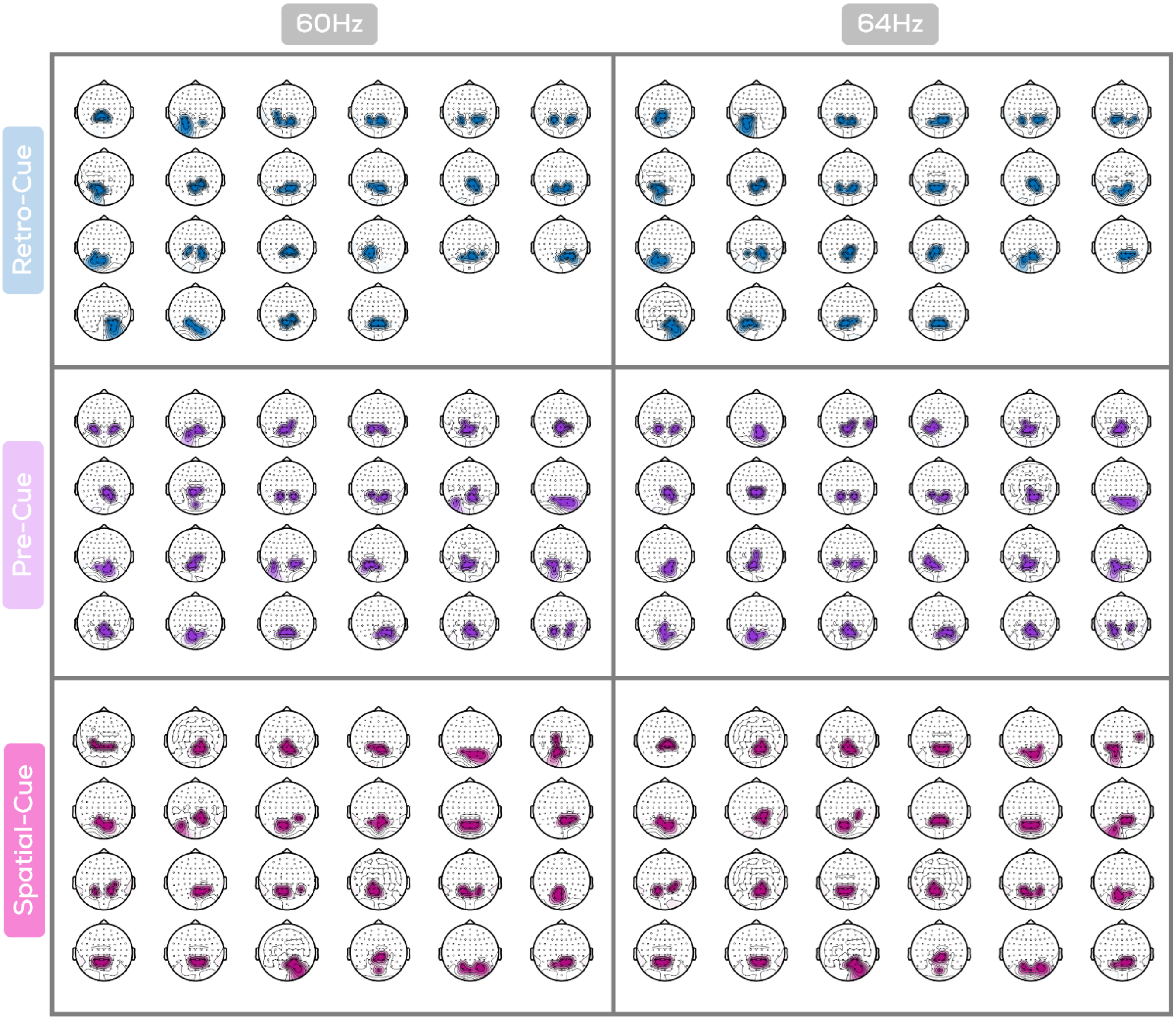

Figure S.1: Overview of selected channels across participants for the retro-Cue, pre-Cue, and spatial Cue experiments. The RIFT responses depicted in Figure 3 and 4 of the main manuscript are computed from 6 selected EEG channels yielding the strongest overall RIFT coherence for each participant, and each frequency. These topographies provide an exhaustive overview of selected channels.

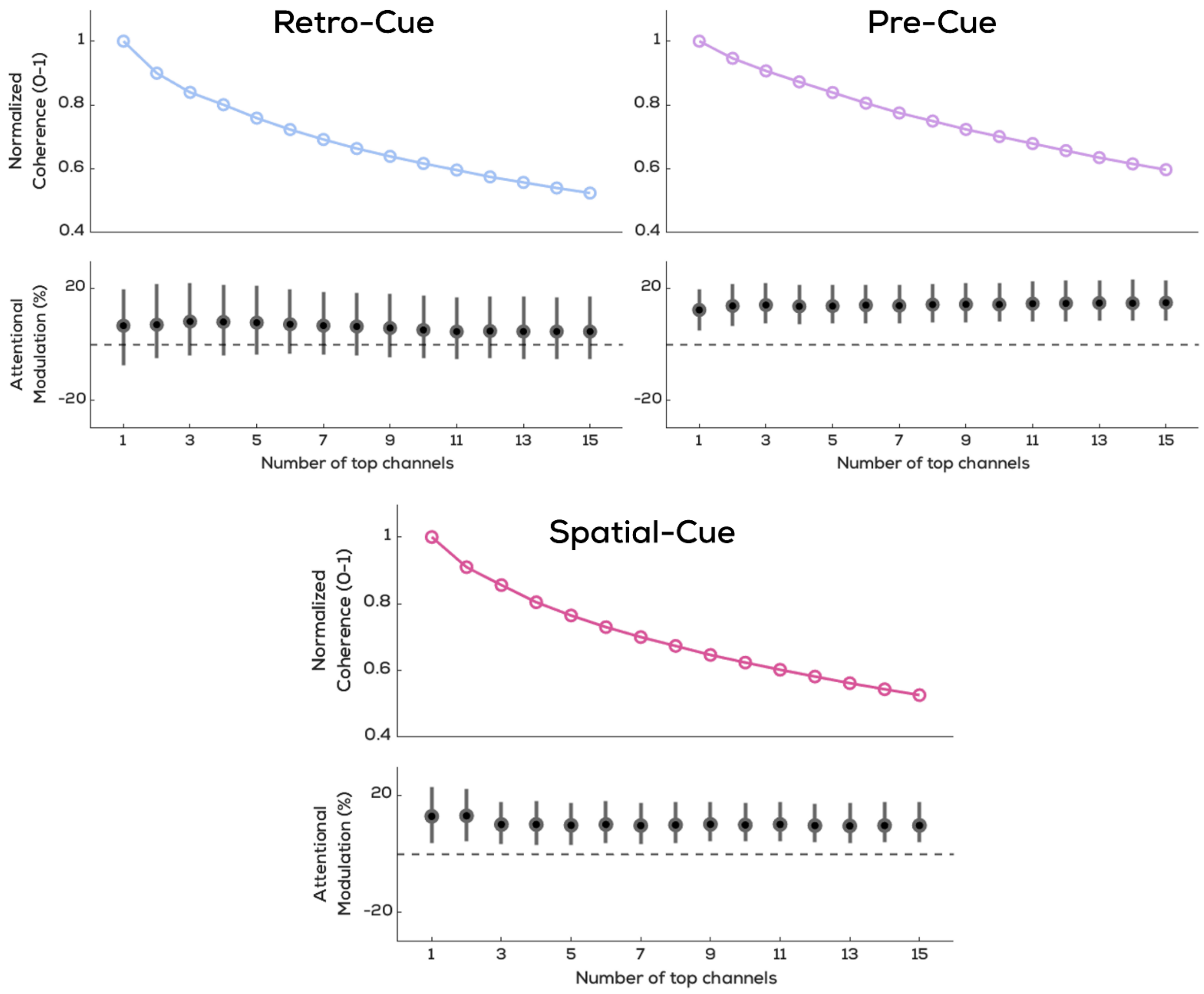

Figure S.2: Mean coherence and attentional modulation in pre and retro-cue tasks as a function of N-averaged channels. In Figure 4 of the manuscript, we report coherence measured from the individual top 6 channels showing strongest coherence averaged across all trials. Here, we show that our conclusions about the attentional modulation of the RIFT response are independent of the number of averaged channels. Although mean coherence decreases as we select more channels (top row), the attentional modulation remains consistent in both pre and retro-cue experiments (top 1 to 15 channels, bottom row) when expressed as a fraction of the mean coherence.

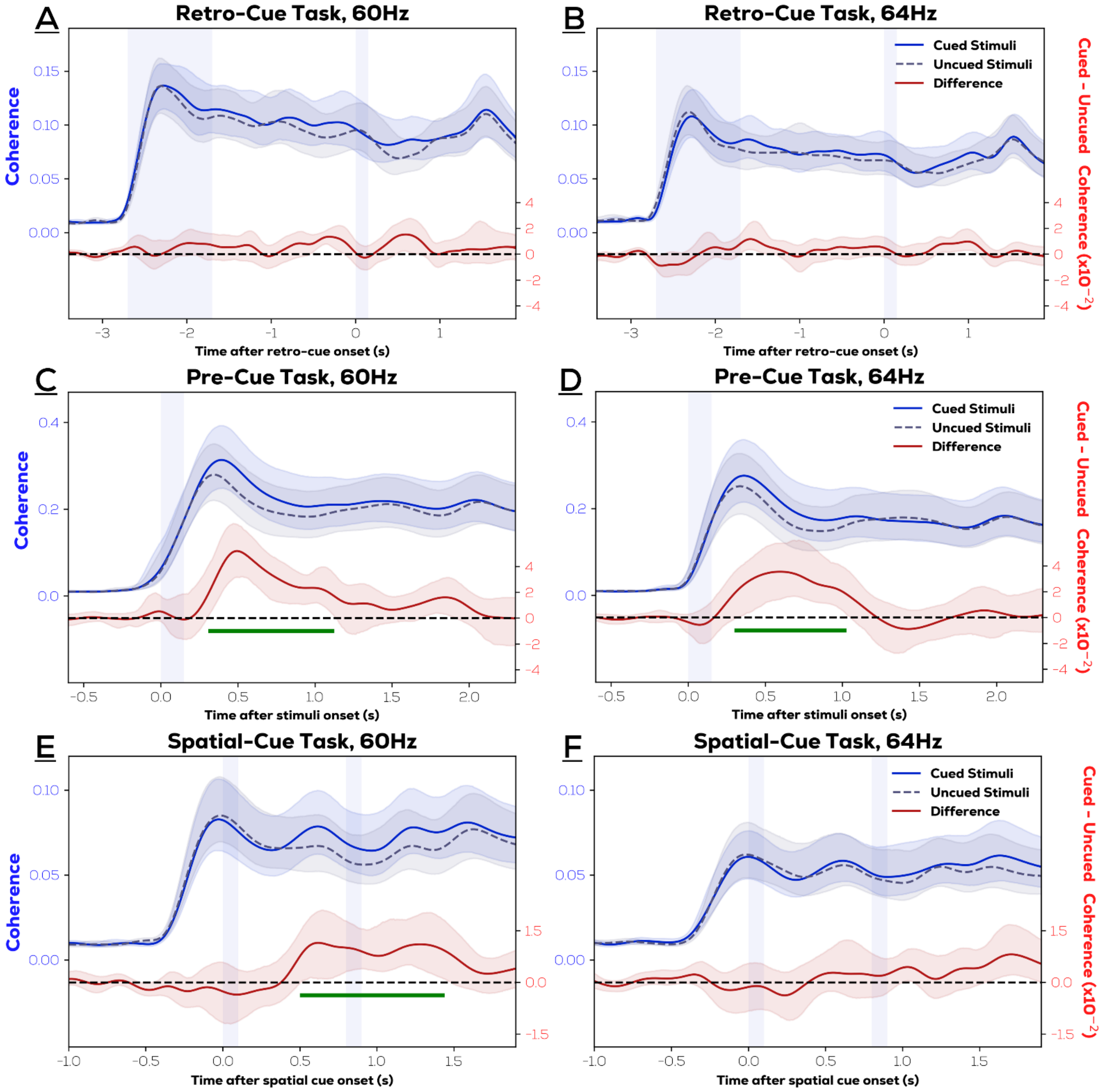

Figure S.3: Frequency-wise RIFT attentional modulation. Extension to Figures 4 and 8B, showing that the key effect reported in the main manuscript persists when separately analyzing responses to 60Hz and 64Hz tagged items. Here, we show the same figures for each task separated by frequency. RIFT coherence from frequencies corresponding to cued vs. uncued stimuli (blue) and difference (red) in the **A-B:** retro-cue task, **C-D:** pre-cue task, and **E-F:** spatial-cue task (shaded region - 95% bootstrapped CIs).

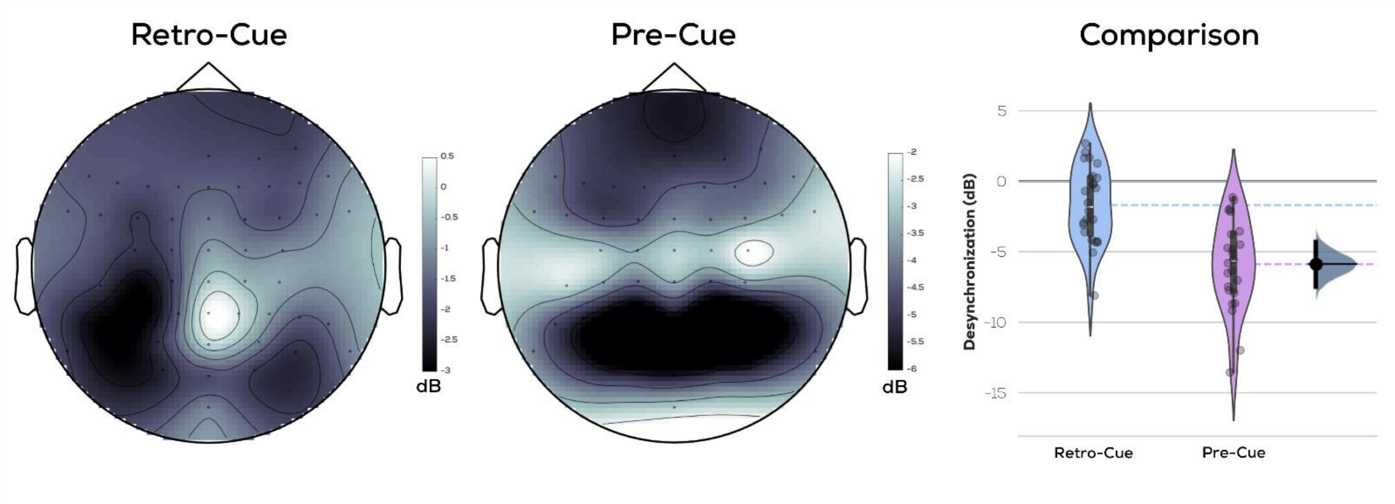

Figure S.4: In Figure 5 of the manuscript we report lateralized alpha as a neural marker of spatial layouts in internal and external attention. Here, we show the alpha desynchronization – the average drop in alpha power (8-13.5Hz) upon presentation of retro-cue/stimuli – that was subtracted between left- and right-cued trials to produce the alpha lateralization, and its comparison across tasks using a permutation test of mean differences. (shaded patch indicates kernel density estimation of respective scatterplots; dashed lines indicate respective means; gray indicates distribution of bootstrapped mean differences; black bar indicates 95% CIs). The pre-cue task showed a considerably stronger decrease in alpha power (mean difference = -4.21, 95% bootstrapped CIs of mean differences = [-5.88, -2.61]).

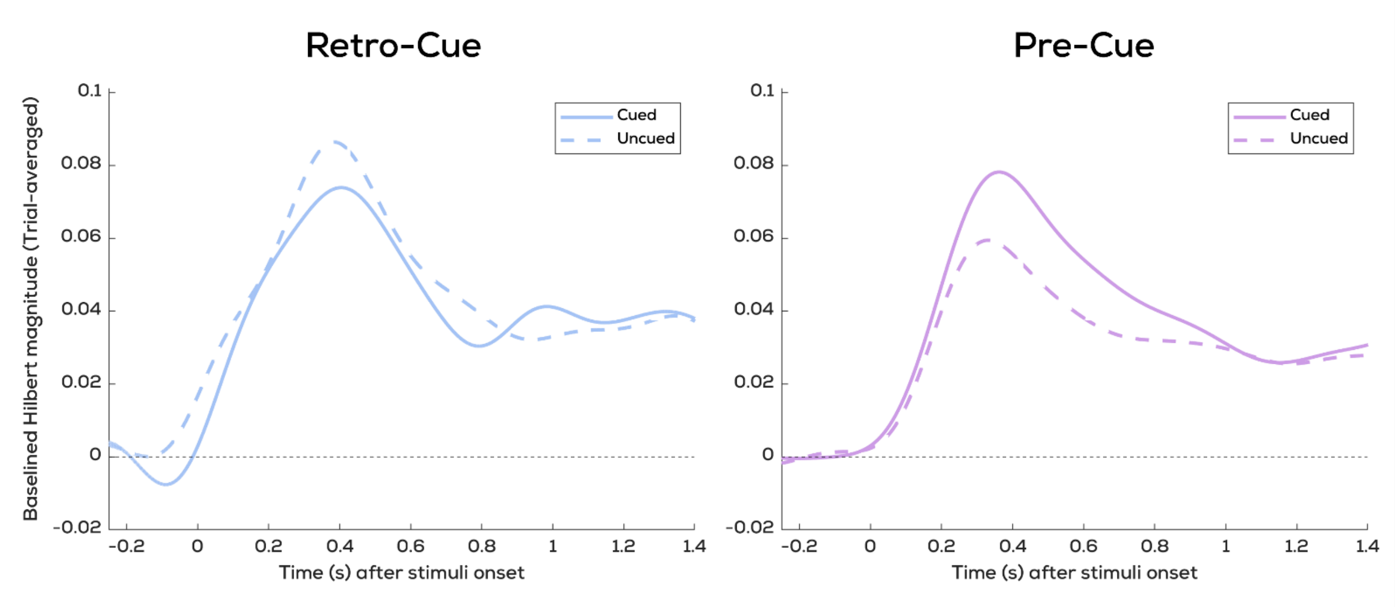

Figure S.5: Average trial-wise hilbert magnitudes are equivalent between the retro-cue and pre-cue experiment. In Figure 4 of the manuscript, we report differences between the retro-cue and pre-cue experiments as measured by coherence. However, the magnitude of coherence is considerably different across the two experiments. In Figure 7 of the manuscript, we replicate these findings through linear mixed modeling using another metric; trial-wise Hilbert magnitudes. Here, we present the average Hilbert magnitudes for the retro-cue and pre-cue experiments, showing that the two are equivalent in amplitude..

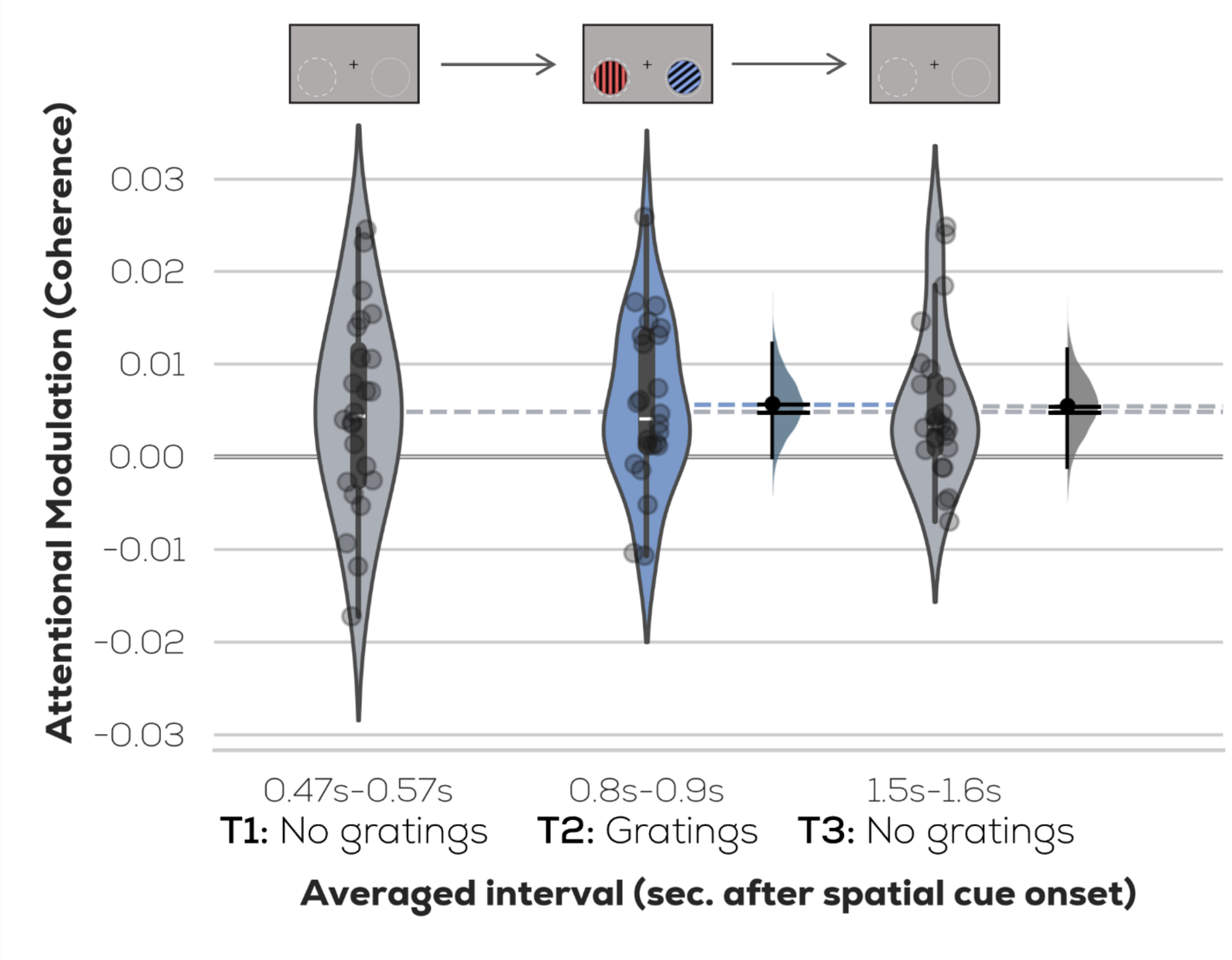

Figure S.6: Attentional RIFT modulation in the spatial cue experiment is consistent across periods of time with and without visible gratings. The objective of the spatial cue experiment was to show that perceptual input is not necessary to evoke an attentional RIFT modulation. In Figure 8B of the manuscript, we confirm this by showing that we measure a stronger RIFT response from the cued location compared to the uncued location already before any gratings are present on the screen. Here we extend this point by comparing the attentional modulations of Figure 8B across three 100ms intervals: (i) the initial onset of this attentional modulation (0.47-0.57s), (ii) the average 100ms display interval of the gratings (0.80-0.90s), and (iii) a late interval a few hundred milliseconds after the grating onset (1.50-1.60s).

A permutation test of mean differences showed no difference between the initial time interval T1 and T2 (mean difference = 0.00075, 95% bootstrapped CIs of mean differences = [-0.00384, 0.00590]) or T3 (mean difference = 0.00054, 95% bootstrapped CIs of mean differences = [-0.00472, 0.00531]). Shaded patches indicate kernel density estimations of respective scatterplots; dashed lines indicate respective means; sideways distributions indicate bootstrapped mean differences between the earliest and the corresponding timepoint; black bar indicates 95% CIs.

The attentional modulation of the RIFT response itself was observed in all three time intervals (bootstrapped 95% confidence intervals, p-values; T1: [0.0008, 0.0091], p=0.0108; T2: [0.0022, 0.0090], p=0.0008; T3: [0.0027, 0.0093], p=0.0002).
